# Evaluation of a Set of C9 *N*-acyl Neu5Ac2en Mimetics as Viral Sialidase Selective Inhibitors

**DOI:** 10.1155/2011/539245

**Published:** 2010-12-08

**Authors:** Sadagopan Magesh, Nongluk Sriwilaijaroen, Setsuko Moriya, Hiromune Ando, Taeko Miyagi, Yasuo Suzuki, Hideharu Ishida, Makoto Kiso

**Affiliations:** ^1^Department of Applied Bioorganic Chemistry, Faculty of Applied Biological Sciences, Gifu University, Gifu, Japan; ^2^Department of Medicinal Chemistry, Ernest Mario School of Pharmacy, Rutgers, The State University of New Jersey, USA; ^3^Faculty of Medicine, Thammasat University (Rangsit Campus), Pathumthani, Thailand; ^4^Department of Biomedical Sciences, College of Life and Health Sciences, Chubu University, Aichi, Japan; ^5^Cancer Glycosylation Research, Institute of Molecular Biomembrane and Glycobiology, Tohoku Pharmaceutical University, Sendai, Japan; ^6^Institute for Integrated Cell-Material Sciences (iCeMS), Kyoto University, Japan

## Abstract

Identification of selective influenza viral sialidase inhibitors is highly desirable in order to minimize or avoid the adverse effects due to the possible inhibition of endogenous human sialidases. We recently reported the evaluation of C9 *N*-acyl Neu5Ac2en mimetics as probes for human sialidases. Herein, we describe the *in vitro* activity of the same set of C9 *N*-acyl Neu5Ac2en mimetics against sialidases expressed by influenza virus A/PR/8/34 (H1N1), A/Memphis/1/72 (H3N2), and A/Duck/313/78 (H5N3) strains. Compound **8** is identified as a promising starting point for the development of viral sialidase selective inhibitors. Multiple sequence alignment and molecular docking techniques are also performed to explore the plausible interaction of compound **8** with viral sialidases.

## 1. Introduction

Influenza is a perceivably benign condition that develops in approximately 20% of the world's population and kills 0.25 to 0.5 million people every year worldwide, according to the WHO [1]. Influenza can cause a high level of mortality, particularly in children, elderly, or those with chronic underlying conditions of lung, heart, kidney, and so forth [2]. There have been three influenza pandemics in the 20th century, and this has lead to millions of deaths with the appearance of a new strain of the virus in each pandemic [3, 4]. Since June 11, 2009, a new strain of swine-origin influenza A virus subtype H1N1 has been declared as the first global influenza pandemic of the 21st century. As of July 4, 2010, over 18311 deaths in more than 214 countries have been confirmed (http://www.who.int/csr/don/2010_07_09/en/index.html). Influenza viruses belong to the Orthomyxoviridae family and are divided into three types, namely, A, B, and C. Influenza A virus, in particular, represents a significant health risk to the public due to both its ability to spread rapidly among humans and being associated with major epidemic outbreaks [5].

Influenza virus is an enveloped virus containing eight segmented, single (nonpaired), and negative sense RNA strands that code for 11 proteins [6, 7], including two glycoproteins (hemagglutinin (HA), neuraminidase (NA) (also known as sialidase), two matrix proteins (M1 and M2), two nonstructural (NS) proteins (NS1 and NS2), nucleoprotein (NP), two polymerase basic proteins (PB1 and PB2), polymerase acidic protein (PA), and basic polymerase 1 frame 2 protein (PB1-F2). Viruses of the influenza type A are subtyped based on the HA and NA, antigenic surface glycoproteins found on the viral envelope, which are essential for viral entry and replication in the infective cycle of influenza [8]. There are 16 hemagglutinin (H1 to H16) and 9 neuraminidase subtypes (N1 to N9) that have been identified. Neuraminidases from influenza type A virus form two genetically distinct groups: group-1 containing N1, N4, N5, and N8 subtypes whereas group-2 with N2, N3, N6, N7, and N9 subtypes [9–20]. Sialidase activity of the influenza neuraminidase cleaves the glycosidic linkage to sialic acid (**1**) on host cells and the surface of the viral particles to facilitate the spread of virions in the host [21]. Therefore, sialidase activity is required for the efficient viral release, and inhibition of sialidase activity is proved to be effective in the treatment or prevention of influenza [22].

**Figure 1:**
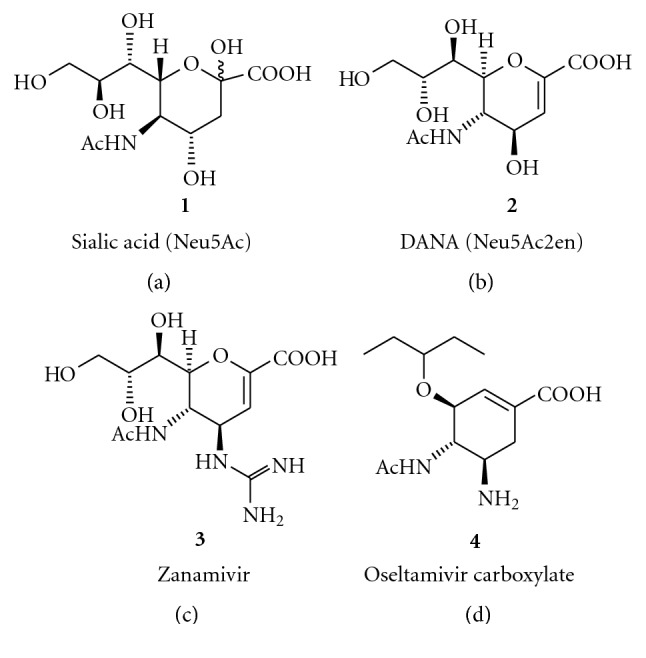
Structures of sialic acid (**1**), DANA (**2**), zanamivir (**3**), and oseltamivir (**4**).

In late 1960s, DANA **2** (5-acetamido-2,6-anhydro-3,5-dideoxy-D-*glycero*- D-*galacto*-non-2-enonic acid, Neu5Ac2-en), a transition-state analogue, is identified as an effective first inhibitor of sialidase enzymes [23]. Using the advantage of structure-based drug design method, two potent sialidase inhibitors, Zanamivir **3** (Relenza, GlaxoSmithKline) and an ester prodrug oseltamivir **4 **(Tamiflu, Gilead/Roche), were designed and developed for the prevention and the treatment of influenza and were complementing the widespread use of influenza vaccines [24, 25] (Figure 1). Both sialidase (NA) enzyme inhibition and X-ray crystallography studies of these inhibitors also suggest that the strategy of designing an inhibitor of NA that binds to the highly conserved active site of the NA achieves the desired goal of activity against all influenza NA subtypes, N1–N9, and influenza B viruses [26, 27]. However, with use of these licensed drugs, several mortalities, severe allergic reactions, and neuropsychiatric events have been reported, particularly in Japan [28]. Also, the FDA has issued a warning label for Tamiflu after reports of serious psychiatric side effects in patients receiving the drug, especially in children [29]. Some of the observed adverse effects have been speculated as a reason of nonselective endogenous human sialidase inhibition by these drugs, although no statistically significant relationship has been established till present [30, 31].

Four types of human sialidases are known and have been classified based on their subcellular localization, namely, the intralysosomal sialidase (NEU1), the cytosolic sialidase (NEU2), the plasma membrane-associated sialidase (NEU3), and the lysosomal or mitochondrial membrane-associated sialidase (NEU4) [32]. These isoforms differ in their substrate specificities, enzymatic properties, and physiological functions. Human sialidases are involved in a wide variety of biological processes through modulating the sialoglycoconjugates [33]. They are implicated in various cellular events such as cell metabolism, cell differentiation, cell growth, and apoptosis including immune functions [34]. Human sialidases, although differ from the viral sialidases in their primary structure and enzymatic properties, show striking similarities in the tertiary structural aspects and active-site architecture [35]. Therefore, active site-directed nonspecific inhibitors of viral sialidase could also potentially inhibit human sialidase isoforms. For instance, inhibition of NEU3 in normal subjects may contribute to the onset of neuropsychiatric symptoms since NEU3 is proved to be an important molecule in the neuronal differentiation [30]. The above mentioned facts suggests that there is a significant need for viral sialidase selective inhibitors with other improved properties (including increased efficacy and reduced sensitivity to resistance) relative to the currently marketed drugs.

**Figure 2:**
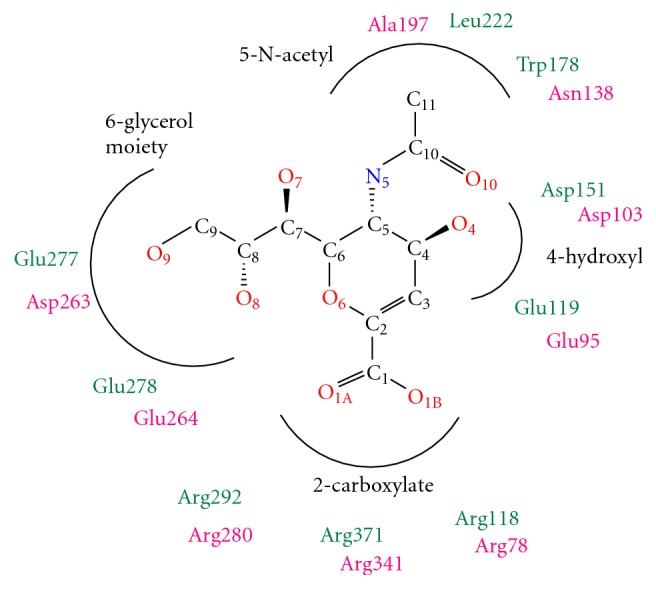
Schematic representation of DANA and its four constituent groups required for binding and conserved residues of NEU1 active site (purple) in comparison with viral sialidase active site (green).

The structural analysis of various sialidase-DANA complexes suggests that four main functional groups that bind to the enzyme active site are 2-carboxylate, 4-hydroxyl, 5-*N-*acetyl, and 6-glycerol moiety (Figure 2) [36]. More importantly, 2-carboxylate (-C_1_O_1A_O_1B_H) group is absolutely necessary for the inhibitory activity. It is well established that the Arg triad in the immediate vicinity of the carboxylate group plays a key role in orienting and stabilizing various inhibitors [37, 38]. In our previous work, we reported a series of amide-linked C9 modified DANA analogues as human sialidase inhibitors, and structure-based methods were used to investigate the observed activity [39]. Results showed that some of the C9 amide-linked hydrophobic analogues of DANA are selective for human lysosomal sialidase (NEU1). The mixed structure and sequence alignment of viral sialidase with human sialidases revealed that NEU1 has a higher sequence similarity (34%) as compared to other isoforms (25–27%), and the active site architecture of NEU1 is more closely related to viral sialidase than other isoforms though striking variations are present [36]. In particular, near C9 of DANA, the conserved E277 of viral sialidase that forms important H-bonds with O8 and O9 hydroxyl groups of DANA is also conserved in NEU1 with a chemically equivalent residue (Asp263), while other isoforms are substituted with nonpolar amino acid residues (Figure 2). Considering this, we planned to evaluate certain C9 DANA amides, (I), against viral sialidases and compared their selectivity over human sialidases. Herein, we report the viral sialidase inhibitory activities and complete experimental details of synthesis and screening of C9 amide-linked hydrophobic analogues.
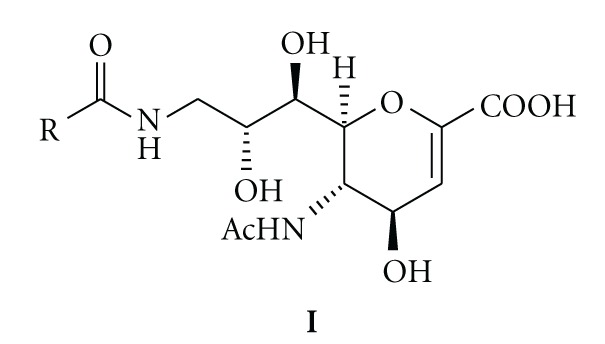
(I)


## 2. Results and Discussion

### 2.1. Sialidase Inhibitory Activity

A set of C9 *N*-acyl Neu5Ac2en derivatives were synthesized according to the procedures previously described. The viral sialidase inhibitory activities of compounds (**5–14**) were evaluated against three different strains of influenza A virus (H1N1, H3N2, and H5N3) using DANA **2 **as a reference compound. Results expressed as 50% inhibitory concentration (IC_50_) values are presented in Table 1 together with the previously reported human sialidase inhibitory activities of compounds (for **2**, **5,** and **8**) [39]. Some compounds demonstrate moderate to good inhibitory activities against viral sialidases with IC_50_ of lower than 100 *μ*M. Moreover, most of the compounds show more activity against sialidase of H1N1 strain over other tested viral strains. The substitution of the small groups by higher homologues at C9 amide position results in a substantial loss of inhibitory activity and might reflect a spatial restriction in the active site of viral sialidases. In particular, the bulky groups of compounds (**6, 7,** and **14**) severely diminish the activity, and this effect becomes more evident with other viral sialidases of H3N2 and H5N3. Indeed, compounds **5** (22 *μ*M for H1N1, 34 *μ*M for H3N2, and 97 *μ*M for H5N3) and **8 **(9 *μ*M for H1N1, 16 *μ*M for H3N2, and 98 *μ*M for H5N3) show IC_50_ values close to DANA (1 *μ*M for H1N1, 9 *μ*M for H3N2, and 11 *μ*M for H5N3) and inhibit the viral sialidases with higher affinity as compared to other compounds in this series. Interestingly, the viral/human sialidase selectivity profiles of compounds **5** (IC_50_ 58 *μ*M for NEU1, >1000 *μ*M for NEU2, >1000 *μ*M for NEU3, and 580 *μ*M for NEU4) and **8 **(680 *μ*M for NEU1, >1000 *μ*M for NEU2, >1000 *μ*M for NEU3, and 825 *μ*M for NEU4) are better than those of DANA (143 *μ*M for NEU1, 43 *μ*M for NEU2, 61 *μ*M for NEU3, and 74 *μ*M for NEU4). These data indicate that compounds **5** and **8** could be further explored for the design of viral sialidase selective inhibitors. 

### 2.2. Molecular Modeling

**Figure 3:**
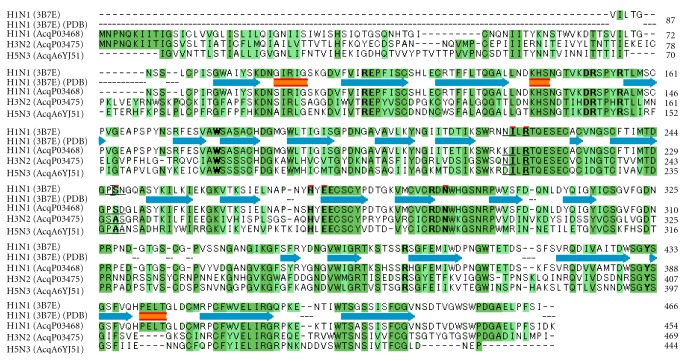
The multiple-sequence alignments of sialidase protein of viral strains (H1N1 [AcqP03468], H3N2 [AcqP03475], and H5N3 [AcqA6YJ51]) and H1N1 (3B7E). Identical and similar residues are shown on a dark green and a light green background, respectively. The active site amino acid residues corresponding to the active site of 3B7E are shown in bold. Amino acid residues interacting with C9 substituent of compound 8 in the active site model of H1N1 (3B7E) are indicated in red. Amino acid differences in the viral sialidases corresponding to the residue Ser246 (3B7E) and its vicinity are underlined.

**Figure 4:**
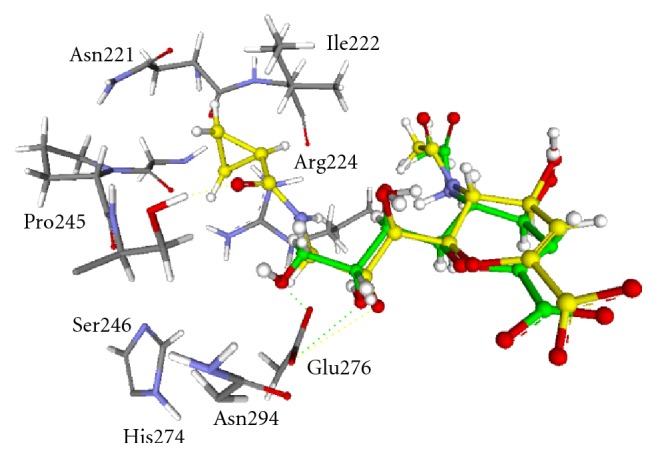
Structural overlap of compound **8** (yellow) and DANA (green) in the active site of H1N1 (3B7E). Dotted lines represent the hydrogen bonding. Active site residues interacting with C9 substituent of compound **8** are only indicated for clarity.

A mixed structural-sequence analysis and molecular docking were performed to explain why there are notable differences in binding affinities and selectivities. The crystal structure of H1N1 (A/Brevig Mission/1/18) (PDB code, 3B7E) was selected as the representative for determining the information regarding spatial disposition discriminating residues [40]. The multiple-sequence alignments of the tested viral strains (H1N1 [AcqP03468], H3N2 [AcqP03475], and H5N3 [AcqA6YJ51]) and 3B7E were performed (Figure 3). The best compound **8** was chosen to be docked into the DANA's binding site of H1N1 (3B7E), and then obtained protein-ligand complex was energetically minimized. We can infer from Figures 3 and 4 that the putative active site residues interacting with compound **8** are highly conserved in all three viral sialidases, except a striking difference at Ser246 (3B7E) and its neighboring residues. This difference is positioned in the vicinity of C-9 of DANA's scaffold and could be a reason for differential interaction of the present series of compounds with their binding regions. Taking a close look at the binding mode of C9 substituent of compound **8** in the active site of H1N1, it can be seen that one out of two conserved H-bond interactions of DANA with Glu (276 in H1N1) is broken and a new H-bond is formed with Ser246 (H1N1). In the case of H3N2 and H5N3, this residue is substituted with a hydrophobic residue Ala that can cause a loss of H-bond and a subsequent drop in the inhibitory activity as compared to H1N1. The cyclopropyl ring of compound **8** interacts with a fleck of hydrophobic surface mainly defined by the side chains of Asn211, Ile222, Arg224, Ser246, and Pro227. As seen in Table 1, the small structural differences at C9 substituents that likely interact with this hydrophobic surface had significant differences in the inhibitory activity. The above mentioned observations indicate that cyclopropyl group can form optimal interactions, and even a slight increase in size of the substituent can probably cause repulsive interactions with this hydrophobic surface. Although the difference is not significant, the slightly increased activity of compound **8** over compound **5** could be due to the conformational rigidity of the cyclopropyl group that is also proposed for its decreased activity against human sialidases as compared to DANA. 

## 3. Conclusion

In summary, this study describes a set of C9 *N*-acyl Neu5Ac2en mimetics as viral sialidase selective inhibitors. The results indicate that the cyclopropyl substituent at C9 shows an optimal inhibitory activity for the current series of compounds and also exhibits a better selectivity profile than the reference compound DANA. Multiple sequence alignment and molecular docking studies give some possible explanations on the interaction of compound **8** with the viral sialidases. We strongly believe that the information derived from present study could be potentially utilized for the design of selective viral sialidase inhibitors over human sialidases and such selective inhibitors are of interest in the context of the future development of anti-influenza agents with minimum or less adverse effects in influenza patients.

## 4. Experimental

### 4.1. The Set of Investigated Compounds

A total of 10 compounds (5-Acetamido-9-acylamido-2,3,5,9-tetra deoxy-D-glycero-D- galacto-non-2-eno-pyranosonic acids) (**5–14**) selected for viral sialidase screening in this study were prepared according to the procedure previously described [39]. (The spectral data of compounds is given in the supplementary section available at doi:10.1155/2011/539245). 

### 4.2. Viral Sialidase Screening

Inhibitory activities of compounds (**5–14**) against sialidase of influenza A viruses were assayed fluorometrically using 4-methylumbelliferyl *N*-acetylneuraminic acid (MUNA) (Toronto Research Chemicals, ON, Canada) as a substrate. One unit of viral sialidase is defined as the enzyme amount required for liberation of 1 nmol of MU per min at 37°C at pH 6.0. The reaction was carried out in a total volume of 15 *μ*L in 20 mM sodium acetate buffer, pH 6.0. Five *μ*L of each serial dilution of compounds (**5–14**) was preincubated with 5 *μ*L of the virus containing 2 enzyme units for 15 min at 37°C. Five *μ*L of MUNA substrate is added into each compound-virus mixture to obtain a final concentration of 1 Km (Michaelis-Menten constant). After incubation for 15 min at 37°C, the reaction was terminated by adding 200 *μ*L of 100 mM sodium carbonate-bicarbonate buffer, pH 10.6. MU products equivalent to NA released were then detected at excitation of 355 nm and emission of 460 nm by a plate reader (Mithras LB940, Berthold Technologies, Pforzheim, Germany). IC_50_ values were determined using nonlinear regression analysis with GraphPad Prism software (San Diego, CA, USA). DANA (**2**, Neu5Ac2en) was also tested for the comparison. 

### 4.3. Molecular Modeling

All computations and simulations were carried out on an Intel P4-based Microsoft windows 2000 workstation using Discovery Studio Modeling 1.5 Package (Accelrys) [41].

#### 4.3.1. Multiple Sequence Alignment

The amino acid sequences of A/PR/8/34 virus [H1N1] (accession no P03468), A/Memphis/1/72 virus [H3N2] (accession no P03475), and A/Duck/313/78 virus [H5N3] (accession no P03468) were obtained from Swiss-Prot database [42], and the sequence of A/Brevig Mission/1/18 virus [H1N1] was extracted from its crystal structure, (PDB code: 3B7E). Multiple alignment process was then carried out on the selected sequences alignments using CLUSTAL W program [43]. The Blosum scoring matrix was selected with a gap penalty of 10 and a gap extension penalty of 0.05. 

#### 4.3.2. Molecular Docking

The model of compound **8** and DANA was built using crystal structure of zanamivir present in the active site of sialidase (PDB code: 37BE). Then compound **8** and DANA were manually docked into the active site of sialidase structure separately. The obtained complexes were then energetically minimized with 500–1000 iterations of ‘in situ ligand minimization algorithm' using the SMART MINIMIZER program. A distance constraint was applied between carboxylic group of inhibitors and conserved Arg triad in the active site. The interactions between compound **8** and active site amino acid residues were analyzed and also compared with DANA's binding mode to investigate the relative differences in interactions for understanding the affinity differences.

## Supplementary Material

The spectral data of investigated compounds (**5**–**14**) are given.

## Figures and Tables

**Table 1:** Chemical structures of compounds **5–14** and their inhibitory activities against sialidase of three different strains of influenza A virus (A/PR/8/34 virus [H1N1], A/Memphis/1/71 virus [H3N2], and A/Duck/313/78 virus [H5N3]).

				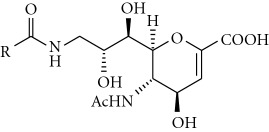				

		Viral sialidase inhibition (IC_50_, *μ*M)^a^			Human sialidase inhibition (IC_50_, *μ*M)^a^			
	R	H1N1	H3N2	H5N3	NEU1	NEU2	NEU3	NEU4

**2**	DANA	1	9	11	143	43	61	74
**5**	Methyl	22	34	97	58	>1000	>1000	580
**6**	Phenyl	>1000	>1000	>1000	—	—	—	—
**7**	Cyclopentyl	850	>1000	>1000	—	—	—	—
**8**	Cyclopropyl	9	16	98	680	>1000	>1000	825
**9**	Propyl	97	559	>1000	—	—	—	—
**10**	2-Methylpropyl	59	>1000	>1000	—	—	—	—
**11**	Isopropyl	54	680	>1000	—	—	—	—
**12**	*n*-Butyl	34	159	>1000	—	—	—	—
**13**	*tert*-Butyl	78	770	>1000	—	—	—	—
**14**	2-Ethylpropyl	698	>1000	>1000	—	—	—	—

^a^IC_50_ values of human sialidases are citied from [39].

## References

[1] World Health Organization http://www.who.int/csr/disease/avian_influenza/en/.

[2] Monto A. S. (2008). Epidemiology of influenza. *Vaccine*.

[3] Cox N. J., Subbarao K. (2000). Global epidemiology of influenza: past and present. *Annual Review of Medicine*.

[4] De Clercq E. (2006). Antiviral agents active against influenza A viruses. *Nature Reviews Drug Discovery*.

[5] Zambon M. C. (1999). Epidemiology and pathogenesis of influenza. *Journal of Antimicrobial Chemotherapy*.

[6] Steinhauer D. A., Skehel J. J. (2002). Genetics of influenza viruses. *Annual Review of Genetics*.

[7] Basler C. F. (2007). Influenza viruses: basic biology and potential drug targets. *Infectious Disorders-Drug Targets*.

[8] Suzuki Y. (2005). Sialobiology of influenza molecular mechanism of host range variation of influenza viruses. *Biological and Pharmaceutical Bulletin*.

[9] Skehel J. (2009). An overview of influenza haemagglutinin and neuraminidase. *Biologicals*.

[10] Russell R. J., Haire L. F., Stevens D. J. (2006). The structure of H5N1 avian influenza neuraminidase suggests new opportunities for drug design. *Nature*.

[11] Collins P. J., Haire L. F., Lin Y. P. (2008). Crystal structures of oseltamivir-resistant influenza virus neuraminidase mutants. *Nature*.

[12] Moscona A. (2005). Neuraminidase inhibitors for influenza. *The New England Journal of Medicine*.

[13] Moscona A. (2009). Global transmission of oseltamivir-resistant influenza. *The New England Journal of Medicine*.

[14] Mihajlovic M. L., Mitrasinovic P. M. (2008). Another look at the molecular mechanism of the resistance of H5N1 influenza A virus neuraminidase (NA) to oseltamivir (OTV). *Biophysical Chemistry*.

[15] Mihajlović M. L., Mitrašinović P. M. (2009). Some novel insights into the binding of oseltamivir and zanamivir to H5N1 and N9 influenza virus neuraminidases: a homology modeling and flexible docking study. *Journal of the Serbian Chemical Society*.

[16] Mihajlovic M. L., Mitrasinovic P. M. (2009). Applications of the ArgusLab4/AScore protocol in the structure-based binding affinity prediction of various inhibitors of group-1 and group-2 influenza virus neuraminidases (NAs). *Molecular Simulation*.

[17] Wang M. Z., Tai C. Y., Mendel D. B. (2002). Mechanism by which mutations at His274 alter sensitivity of influenza A virus N1 neuraminidase to oseltamivir carboxylate and zanamivir. *Antimicrobial Agents and Chemotherapy*.

[18] Du Q. S., Wang S. Q., Chou K. C. (2007). Analogue inhibitors by modifying oseltamivir based on the crystal neuraminidase structure for treating drug-resistant H5N1 virus. *Biochemical and Biophysical Research Communications*.

[19] Mitrasinovic P. M. (2009). On the structure-based design of novel inhibitors of H5N1 influenza A virus neuraminidase (NA). *Biophysical Chemistry*.

[20] Mitrasinovic P. M. (2010). Advances in the structure-based design of the influenza a neuraminidase inhibitors. *Current Drug Targets*.

[21] Matrosovich M. N., Matrosovich T. Y., Gray T., Roberts N. A., Klenk H. D. (2004). Neuraminidase is important for the initiation of influenza virus infection in human airway epithelium. *Journal of Virology*.

[22] von Itzstein M. (2007). The war against influenza: discovery and development of sialidase inhibitors. *Nature Reviews Drug Discovery*.

[23] Meindl P., Tuppy H. (1969). 2-deoxy-2,3-dehydrosialic acids. II. Competitive inhibition of Vibrio cholerae neuraminidase by 2-deoxy-2,3-dehydro-N-acylneuraminic acids. *Hoppe-Seyler's Zeitschrift fur Physiologische Chemie*.

[24] von Itzstein M., Dyason J. C., Oliver S. W. (1996). A study of the active site of influenza virus sialidase: an approach to the rational design of novel anti-influenza drugs. *Journal of Medicinal Chemistry*.

[25] Lew W., Chen X., Kim C. U. (2000). Discovery and development of GS 4104 (oseltamivir): an orally active influenza neuraminidase inhibitor. *Current Medicinal Chemistry*.

[26] Govorkova E. A., Leneva I. A., Goloubeva O. G., Bush K., Webster R. G. (2001). Comparison of efficacies of RWJ-270201, zanamivir, and oseltamivir against H5N1, H9N2, and other avian influenza viruses. *Antimicrobial Agents and Chemotherapy*.

[27] Roberts N. A., Govorkova E. A., Mitrasinovic P. M. (2009). The activity of neuraminidase inhibitor oseltamivir against all subtypes of influenza viruses. *Global View of the Fight against Influenza*.

[28] Hama R. (2008). Fatal neuropsychiatric adverse reactions to oseltamivir: case series and overview of causal relationships. *International Journal of Risk and Safety in Medicine*.

[29] USA Today (2006). *FDA Adds 'Abnormal Behavior' Precaution to Tamiflu Label*.

[30] Hata K., Koseki K., Yamaguchi K. (2008). Limited inhibitory effects of oseltamivir and zanamivir on human sialidases. *Antimicrobial Agents and Chemotherapy*.

[31] Li C. Y., Yu Q., Ye Z. Q. (2007). A nonsynonymous SNP in human cytosolic sialidase in a small Asian population results in reduced enzyme activity: potential link with severe adverse reactions to oseltamivir. *Cell Research*.

[32] Miyagi T., Kato K., Ueno S., Wada T. (2004). Aberrant expression of sialidase in cancer. *Trends in Glycoscience and Glycotechnology*.

[33] Schauer R. (1982). Chemistry, metabolism, and biological functions of sialic acids. *Advances in Carbohydrate Chemistry and Biochemistry*.

[34] Achyuthan K. E., Achyuthan A. M. (2001). Comparative enzymology, biochemistry and pathophysiology of human exo-*α*-sialidases (neuraminidases). *Comparative Biochemistry and Physiology B*.

[35] Chavas L. M. G., Tringali C., Fusi P. (2005). Crystal structure of the human cytosolic sialidase Neu2: evidence for the dynamic nature of substrate recognition. *Journal of Biological Chemistry*.

[36] Magesh S., Suzuki T., Miyagi T., Ishida H., Kiso M. (2006). Homology modeling of human sialidase enzymes NEU1, NEU3 and NEU4 based on the crystal structure of NEU2: hints for the design of selective NEU3 inhibitors. *Journal of Molecular Graphics and Modelling*.

[37] Wang T., Wade R. C. (2001). Comparative binding energy (COMBINE) analysis of influenza neuraminidase-inhibitor complexes. *Journal of Medicinal Chemistry*.

[38] Ortiz A. R., Pisabarro M. T., Gago F., Wade R. C. (1995). Prediction of drug binding affinities by comparative binding energy analysis. *Journal of Medicinal Chemistry*.

[39] Magesh S., Moriya S., Suzuki T., Miyagi T., Ishida H., Kiso M. (2008). Design, synthesis, and biological evaluation of human sialidase inhibitors. Part 1: selective inhibitors of lysosomal sialidase (NEU1). *Bioorganic and Medicinal Chemistry Letters*.

[40] Xu X., Zhu X., Dwek R. A., Stevens J., Wilson I. A. (2008). Structural characterization of the 1918 influenza virus H1N1 neuraminidase. *Journal of Virology*.

[41] *DS Modeling v1.50*.

[42] Bairoch A., Apweiler R. (1997). The SWISS-PROT protein sequence database: its relevance to human molecular medical research. *Journal of Molecular Medicine*.

[43] Thompson J. D., Higgins D. G., Gibson T. J. (1994). CLUSTAL W: improving the sensitivity of progressive multiple sequence alignment through sequence weighting, position-specific gap penalties and weight matrix choice. *Nucleic Acids Research*.

