# Supplementary material for: Evaluation of a Set of C9 N-acyl Neu5Ac2en Mimetics as Viral Sialidase Selective Inhibitors
Source: Int J Med Chem. 2010 Dec 8;2011:539245. doi: 10.1155/2011/539245 (PMC4971745; doi:10.1155/2011/539245)
Supplement: Supplementary file 1 — The spectral data of investigated compounds (5–14) are given. [file 539245.f1.pdf]

Supplementary material

Evaluation of a set of C9 *N*-acyl Neu5Ac2en mimetics as viral sialidase selective inhibitors

Sadagopan Magesh[a *†], Nongluk Sriwilaijaroen[b,c], Setsuko Moriya[d], Hiromune Ando[a], Taeko Miyagi[d], Yasuo Suzuki[c], Hideharu Ishida[a], Makoto Kiso[a,e*]

[a] *Department of Applied Bioorganic Chemistry, Faculty of Applied Biological Sciences, Gifu University, Japan*

[b] *Faculty of Medicine, Thammasat University (Rangsit Campus), Pathumthani, Thailand*

[c] *Department of Biomedical Sciences, College of Life and Health Sciences, Chubu University, Japan*

[d] *Department of Biochemistry, Miyagi Cancer Center Research Institute, Japan*

[e] *Institute for Integrated Cell-Material Sciences (iCeMS), Kyoto University, Japan*

Spectral data of compounds 5 to 14

All NMR spectra were recorded at 400, 500 or 600 MHz. The chemical shifts are reported in δ (ppm) and are relative to the central peak of the solvent which was $CDCl_3$, CD_3OD or D_2O. In order to confirm assignments, 2D NMR experiments were performed using COSY for proton-proton interactions, HMQC and HMBC for experiment for proton–carbon interactions. Mass spectral data were obtained on a Bruker Daltonics microTOF-II in the negative ion detection mode.

5,9-Diacetamido-2,3,5,9-tetradeoxy-D-glycero-D-galacto-non-2-eno-pyranosonic acid (5)

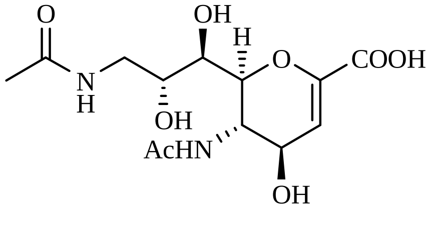

^{1}H NMR (CD_3OD) δ 1.87, 1.93 (6H, 2 × s, 2 × NAc), 3.10 to 3.15 (1H, m, H-9[a]), 3. 29 (1H, dd, $J_{7,8}$ 8.9 Hz, $J_{7,6}$ <1 Hz, H-7), 3.55 (1H, dd, $J_{9b,8}$ 3.1 Hz, $J_{9b,9a}$ 13.7 Hz, H-9[b]), 3.78 to 3.82 (1H, m, H-8), 3.88 (1H, dd, $J_{5,6}$ 10.9 Hz, $J_{5,4}$ 8.9 Hz, H-5), 4.01 (1H, dd, $J_{6,5}$ 10.9 Hz, $J_{6,7}$ <1 Hz, H-6), 4.27 (1H, dd, $J_{4,3}$ 2.1 Hz, $J_{4,5}$ 8.9 Hz, H-4), 5.57 (1H, d, $J_{3,4}$ 2.1 Hz, H-3); ^{13}C NMR ($CDCl_3$) δ 22.7, 22.9 (NC(O)*Me*), 44.6 (C-9),

52.1 (C-5), 68.8 (C4), 70.26 (C7) 72.1 (C8), 77.3 (C6), 108.5 (C3), 150.2 (C2), 170.2 (*C*OOH), 173.9, 174.9 (2 × N*C*(O)Me); HR-MS (Negative): Calcd. For $C_{13}H_{19}N_2O_8$ $(M\text{-}H)^-$, 331.1147, Found 331.1171.

5-Acetamido-9-benzamido-2,3,5,9-tetradeoxy-D-glycero-D-galacto-non-2-eno-pyranosonic acid (6)

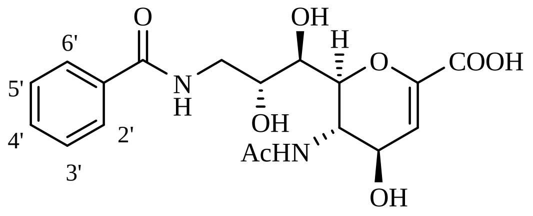

^{1}H NMR (CD_3OD) δ 1.91 (3H, s, NAc), 3.38 (1H, dd, $J_{7,8}$ 9.1 Hz, $J_{7,6}$ <1 Hz, H-7), 3.44 to 3.51 (1H, m, H-9^a), 3.71 (1H, dd, $J_{9b,8}$ 3.2 Hz $J_{9b,9a}$ 13.7 Hz, H-9^a), 3.90 to 3.98 (2H, m, H-5, H-8), 4.08 (1H, dd, $J_{6,5}$ 11.0 Hz, $J_{6,7}$ <1 Hz, H-6), 4.28 (1H, dd, $J_{4,3}$ 2.2 Hz, $J_{4,5}$ 8.6 Hz, H-4), 5.58 (1H, d, $J_{3,4}$ 2.2 Hz, H-3), 7.37 to 7.44 (3H, m, H-3′, H-4′, H-5′), 7.74 to 7.76 (2H, m, H-2′, H-6′); ^{13}C NMR ($CDCl_3$) δ 22.9 (NC(O)*Me*), 45.2 (C-9), 52.3 (C-5), 68.9 (C4), 70.26 (C7) 72.3 (C8), 77.3 (C6), 108.2 (C3), 128.5 (C2′, C-6′), 129.7 (C3′, C-5′), 132.7 (C4′), 136.0 (C1′), 150.5 (C2), 167.8 (N*C*(O) phenyl), 170.5 (*C*OOH), 174.8 (N*C*(O)Me); HR-MS (Negative): Calcd. For $C_{18}H_{21}N_2O_8$ $(M\text{-}H)^-$, 393.1303, Found 393.1347.

5-Acetamido-9-cyclopentanecarboxamido-2,3,5,9-tetradeoxy-D-glycero-D-galacto-non-2-eno-pyranosonic acid (7)

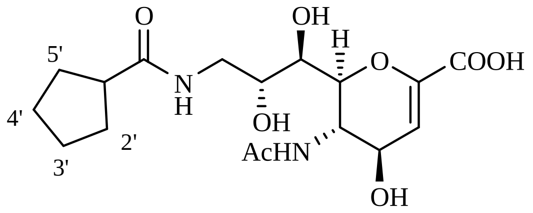

^{1}H NMR (CD_3OD) δ 1.42 to 1.82 (8H, m, H-2′,H-3′, H-4′, H-5′), 1.94 (3H, s, NAc), 2.59 (1H, p, H-1′), 3.23 (1H, H-9^{a,} mixed with C*D3*OD peak), 3.31 (1H, dd, $J_{7,8}$ 9.1 Hz, $J_{7,6}$ <1 Hz, H-7), 3.51 (1H, dd, $J_{9b,8}$ 3.2 Hz, $J_{9b,9a}$ 13.7 Hz, H-9^b), 3.80 to 3.86 (1H, m, H-8), 3.90 (1H, dd, $J_{5,4}$ 8.6 Hz, $J_{5,6}$ 10.5 Hz, H-5), 4.05 (1H, dd, $J_{6,5}$ 10.5 Hz, $J_{6,7}$ <1 Hz, H-6), 4.28 (1H, dd, $J_{4,3}$ 1.8 Hz, $J_{4,5}$ 8.7 Hz, H-4), 5.63 (1H, d, $J_{3,4}$ 1.8 Hz, H-3); ^{13}C NMR ($CDCl_3$) δ 22.9 (NC(O)*Me*), 27.2 (C3′, C4′), 31.8, 31.7 (C2′, C5′), 44.7 (C-9), 46.76 (C1′), 52.3 (C-5), 68.8 (C4), 70.36 (C7) 71.9 (C8), 77.4 (C6), 109.2 (C3), 149.6 (C2), 167.8 (*C*OOH), 174.8 (N*C*(O)Me), 180.1 (N*C*(O) cyclopentyl);

HR-MS (Negative): Calcd. For $C_{17}H_{25}N_2O_8$ $(M-H)^-$, 385.1611, Found 385.1673.

5-Acetamido-9-cyclopropanecarboxamido-2,3,5,9-tetradeoxy-D-glycero-D-galacto-non-2-eno-pyranosonic acid (8)

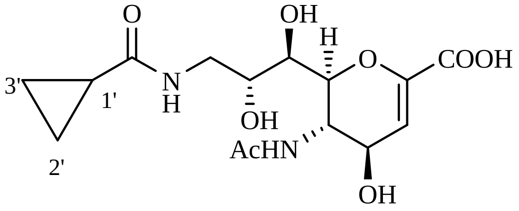

^{1}H NMR (CD_3OD) δ 0.65 to 0.79 (4H, 2 × m, H-2′,H-3′), 1.52 to 158 (1H, m, H-1′), 1.95 (3H, s, NAc), 3.23 to 3.27 (1H, m, H-9[a]), 3.34 (1H, dd, $J_{7,8}$ 8.9 Hz, $J_{7,6}$ <1 Hz, H-7), 3.53 (1H, dd, $J_{9b,8}$ 3.4 Hz, $J_{9b,9a}$ 13.7 Hz, H-9[b]), 3.83 to 3.86 (1H, m, H-8), 3.91 (1H, dd, $J_{5,4}$ 8.9 Hz, $J_{5,6}$ 10.9 Hz, H-5), 4.08 (1H, dd, $J_{6,5}$ 10.9 Hz, $J_{6,7}$ <1 Hz, H-6), 4.31 (1H, dd, $J_{4,3}$ 2.1 Hz, $J_{4,5}$ 8.9 Hz, H-4), 5.73 (1H, d, $J_{3,4}$ 2.1 Hz, H-3); ^{13}C NMR ($CDCl_3$) δ 7.5, 7.4 (C2′, C3′), 15.0 (C1′), 22.9 (NC(O)*Me*), 44.9 (C-9), 52.2 (C-5), 68.5 (C4), 70.5 (C7) 71.6 (C8), 77.6 (C6), 111.1 (C3), 147.8 (C2), 167.9 (*C*OOH), 174.9 (N*C*(O)Me), 177.5 (N*C*(O) cyclopropyl); HR-MS (Negative): Calcd. For $C_{15}H_{21}N_2O_8$ $(M-H)^-$, 357.1303, Found 357.1352.

5-Acetamido-9-butyramido-2,3,5,9-tetradeoxy-D-glycero-D-galacto-non-2-eno-pyranosonic acid (9)

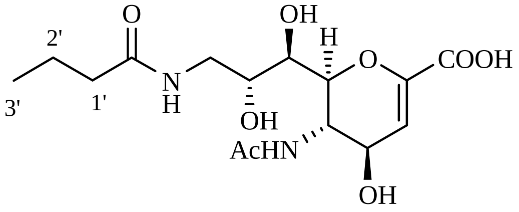

^{1}H NMR (CD_3OD) δ 0.87 (3H, t, H-3′), 1.55 (2H, p, H-2′), 1.94 (3H, s, NAc), 2.12 (2H, t, H-1′), 3.22 to 3.27 (1H, m, H-9[a]), 3.35 (1H, dd, $J_{7,8}$ 8.9 Hz, $J_{7,6}$ <1 Hz, H-7), 3.51 (1H, dd, $J_{9b,8}$ 2.7 Hz, $J_{9b,9a}$ 13.7 Hz, H-9[b]), 3.83 to 3.86 (1H, m, H-8), 3.91 (1H, dd, $J_{5,4}$ 8.9 Hz, $J_{5,6}$ 10.9, H-5), 4.08 (1H, dd, $J_{6,5}$ 10.9 Hz, $J_{6,7}$ <1 Hz, H-6), 4.31 (1H, dd, $J_{4,3}$ 2.7 Hz, $J_{4,5}$ 8.9 Hz, H-4), 5.72 (1H, d, $J_{3,4}$ 2.7 Hz, H-3); ^{13}C NMR (CD_3OD) δ 14.2 (C3′), 20.6 (C2′), 22.9 (NC(O)*Me*), 39.18 (C1′), 44.4 (C-9), 52.2 (C-5), 68.2 (C4), 70.5 (C7) 71.6 (C8), 77.6 (C6), 111.8 (C3), 148.0 (C2), 168.2 (*C*OOH), 174.9 (N*C*(O)Me), 177.1 (N*C*(O) propyl); HR-MS (Negative): Calcd. For $C_{15}H_{23}N_2O_8$ $(M-H)^-$, 359.1460, Found 359.1482.

5-Acetamido-9-(2-methylbutanamido)-2,3,5,9-tetradeoxy-D-glycero-D-galacto-non-2-eno-pyranosonic acid (10)

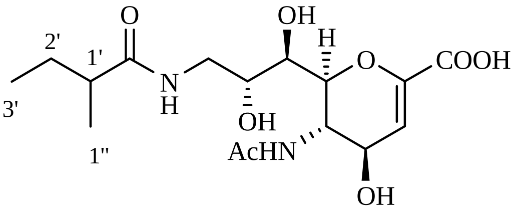

^{1}H NMR (CD_3OD) δ 0.82 (3H, t, H-3′), 1.02 (3H, dd, H-1″ isomers), 1.32, 1.52 (2H, m, H-2′ isomers), 1.93 (3H, s, NAc), 2.16 to 2.22 (1H, m, H-1′), 3.22 to 3.34 (2H, m, H-7, H-9[a]), 3.51 (1H, dd, $J_{9b,8}$ 2.7 Hz, $J_{9b,9a}$ 13.7, Hz, H-9[b]), 3.81 to 3.86 (1H, m, H-8), 3.90 (1H, dd, $J_{5,4}$ 8.9 Hz, $J_{5,6}$ 10.9 Hz, H-5), 4.07 (1H, dd, $J_{6,5}$ 10.9 Hz, $J_{6,7}$ <1 Hz, H-6), 4.30 (1H, dd, $J_{4,3}$ 2.1 Hz, $J_{4,5}$ 8.9 Hz, H-4), 5.69 (1H, d, $J_{3,4}$ 2.1 Hz, H-3); ^{13}C NMR (CD_3OD) δ 12.5 (C-3′), 18.2 (C1″), 22.9 (NC(O)*Me*), 27.5 (C-2′), 39.18 (C1′), 44.4 (C-1′), 44.8 (C-9), 52.4 (C-5), 68.6 (C4), 70.4 (C7) 71.7 (C8), 77.6 (C6), 110.3 (C3), 148.5 (C2), 168.5 (*C*OOH), 174.8 (N*C*(O)Me), 180.5 (N*C*(O) 2-methylpropyl); HR-MS (Negative): Calcd. For $C_{16}H_{25}N_2O_8$ $(M\text{-}H)^-$, 373.1611, Found 373.1702.

5-Acetamido-9-isobutyramido-2,3,5,9-tetradeoxy-D-glycero-D-galacto-non-2-eno-pyranosonic acid (11)

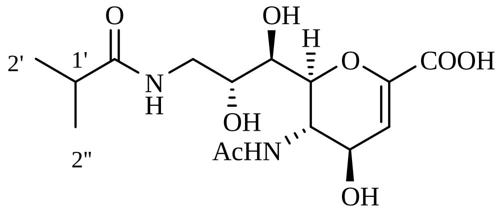

^{1}H NMR (CD_3OD) δ 1.04 (3H, d, H-2′, H-2″), 1.93 (3H, s, NAc), 2.41 (1H, p, H-1′), 3.28 (1H, dd, $J_{9a,8}$ 6.4 Hz, $J_{9a,9b}$ 13.7 Hz, H-9[a]), 3.44 (1H, dd, $J_{7,8}$ 8.9 Hz, $J_{7,6}$ <1 Hz, H-7) 3.49 (1H, dd, J_{9b8} 3.4 Hz, $J_{9b,9a}$ 13.7 Hz, H-9[b]), 3.83 to 3.87 (1H, m, H-8), 3.91 (1H, dd, $J_{5,4}$ 8.9 Hz, $J_{5,6}$ 10.9 Hz, H-5), 4.11 (1H, dd, $J_{6,5}$ 10.9 Hz, $J_{6,7}$ <1 Hz, H-6), 4.34 (1H, dd, $J_{4,3}$ 2.1 Hz, $J_{4,5}$ 8.9 Hz, H-4), 5.85 (1H, d, $J_{3,4}$ 2.1 Hz, H-3)); ^{13}C NMR (CD_3OD) δ 20.1 (C2′, C2″), 22.9 (NC(O)*Me*), 36.4 (C-1′), 44.5 (C-9), 52.2 (C-5), 68.2 (C4), 70.3 (C7) 71.6 (C8), 77.9 (C6), 113.1 (C3), 146.0 (C2), 166.0 (*C*OOH), 175.0 (N*C*(O)Me), 181.3 (N*C*(O) isopropyl); HR-MS (Negative): Calcd. For $C_{15}H_{23}N_2O_8$ $(M\text{-}H)^-$, 359.1460, Found 528.1783.

5-Acetamido-9-pentanamido-2,3,5,9-tetradeoxy-D-glycero-D-galacto-non-2-eno-py

ranosonic acid (12)

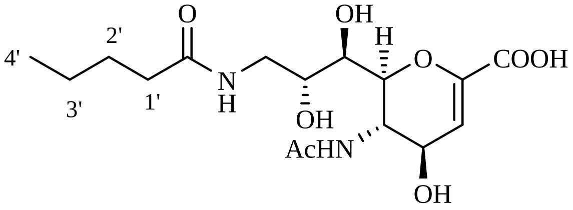

^{1}H NMR (CD_3OD) δ 0.85 (3H, t, H-4′), 1.27 (2H, sex, H-3′), 1.51 (2H, p, H-2′), 1.95 (3H, s, NAc), 2.14 (2H, t, H-1′), 3.23 (1H, H-9^{a}, mixed with C*D3*OD peak), 3.33 (1H, dd, $J_{7,8}$ 8.9 Hz, $J_{7,6}$ <1 Hz, H-7), 3.52 (1H, dd, $J_{9b,8}$ 3.4 Hz, $J_{9b,9a}$ 13.7 Hz, H-9^{b}), 3.82 to 3.86 (1H, m, H-8), 3.90 (1H, dd, $J_{5,4}$ 8.9 Hz, $J_{5,6}$ 10.3 Hz, H-5), 4.08 (1H, dd, $J_{6,5}$ 10.3 Hz, $J_{6,7}$ <1 Hz, H-6), 4.32 (1H, dd, $J_{4,3}$ 2.0 Hz, $J_{4,5}$ 8.9 Hz, H-4), 5.78 (1H, d, $J_{3,4}$ 2.0 Hz, H-3); ^{13}C NMR (CD_3OD) δ 14.3 (C4′), 22.9 (NC(O)*Me*), 23.6 (C3′), 29.4 (C2′), 37.0 (C1′), 44.5 (C-9), 52.2 (C-5), 68.3 (C4), 70.3 (C7) 71.8 (C8), 77.8 (C6), 112.0 (C3), 147.0 (C2), 167.1 (*C*OOH), 175.0 (N*C*(O)Me), 177.3 (N*C*(O) *n*-butyl); HR-MS (Negative): Calcd. For $C_{16}H_{25}N_2O_8$ $(M-H)^-$, 373.1611, Found 373.1668.

5-Acetamido-9-pivalamido-2,3,5,9-tetradeoxy-D-glycero-D-galacto-non-2-eno-pyranosonic acid (13)

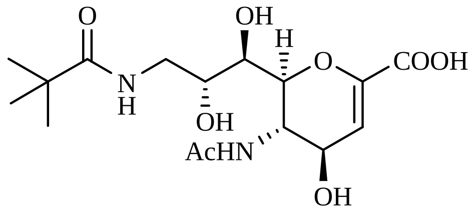

^{1}H NMR (CD_3OD) δ 1.11 (9H, s, NPiv), 1.93 (3H, s, NAc), 3.30 to 3.34 (2H, m, H-7, H-9^{a}), 3.45 (1H, dd, $J_{9b,8}$ 3.3 Hz, $J_{9b,9a}$ 13.6 Hz, H-9^{b}), 3.83 to 3.89 (1H, m, H-8), 3.90 (1H, dd, $J_{5,4}$ 9.0 Hz, $J_{5,6}$ 10.2 Hz, H-5), 4.09 (1H, dd, $J_{6,5}$ 10.2 Hz, $J_{6,7}$ <1 Hz, H-6), 4.32 (1H, dd, $J_{4,3}$ 2.1 Hz, $J_{4,5}$ 8.9 Hz, H-4), 5.78 (1H, d, $J_{3,4}$ 2.1 Hz, H-3); ^{13}C NMR (CD_3OD) δ 21.4 (NC(O)*Me*), 26.5 (C2′, C2″, C2‴), 38.4 (C-1′), 43.3 (C-9), 50.6 (C-5), 66.8 (C4), 69.3 (C7) 70.2 (C8), 76.2 (C6), 110.6 (C3), 145.4 (C2), 167.7 (*C*OOH), 173.4 (N*C*(O)Me), 180.9 (N*C*(O)CMe_3); HR-MS (Negative): Calcd. For $C_{16}H_{25}N_2O_8$ $(M-H)^-$, 373.1611, Found 373.1656.

5-Acetamido-9-(2-ethylbutanamido)-2,3,5,9-tetradeoxy-D-glycero-D-galacto-non-2-eno-pyranosonic acid (14)

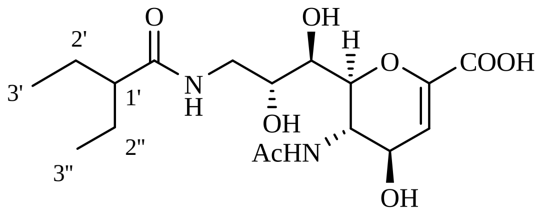

^{1}H NMR (CD_3OD) δ 0.82 (6H, t, H-3′, H-3″), 1.38, 1.48 (4H, 2 × m , H-2′, H-2″), 1.93 (3H, s, NAc), 2.00 (1H, m, H-1′), 3.31 (1H, dd, $J_{9a,8}$ 6.1 Hz, $J_{9a,9b}$ 13.7 Hz, H-9^{a}), 3.35 (1H, dd, $J_{7,8}$ 8.0 Hz, $J_{7,6}$ <1 Hz, H-7) 3.51 (1H, dd, $J_{9b,8}$ 2.7 Hz, $J_{9b,9a}$ 13.7 Hz, H-9^{b}), 3.84 to 3.89 (1H, m, H-8), 3.91 (1H, dd, $J_{5,4}$ 8.9 Hz, $J_{5,6}$ 10.3 Hz, H-5), 4.11 (1H, dd, $J_{6,5}$ 10.3 Hz, $J_{6,7}$ <1 Hz, H-6), 4.31 (1H, dd, $J_{4,3}$ 2.1 Hz, $J_{4,5}$ 8.9 Hz, H-4), 5.73 (1H, d, $J_{3,4}$ 2.1 Hz, H-3); ^{13}C NMR (CD_3OD) δ 12.7, 12.6 (C3′, C3″), 22.9 (NC(O)*Me*), 27.0 (C2′, C2″), 44.7 (C-9), 52.1 (C-1′), 52.2 (C-5), 68.6 (C4), 70.5 (C7), 71.7 (C8), 77.6 (C6), 110.7 (C3), 148.2 (C2), 168.2 (*C*OOH), 174.9 (N*C*(O)Me), 179.9 (N*C*(O) 2-ethylpropyl); HR-MS (Negative): Calcd. For $C_{17}H_{27}N_2O_8$ $(M-H)^-$, 387.1767, Found 387.1798.
